# Highly expressed of BID indicates poor prognosis and mediates different tumor microenvironment characteristics in clear cell renal cell carcinoma

**DOI:** 10.1007/s12672-024-01035-8

**Published:** 2024-05-20

**Authors:** Jiayi Zeng, Chuangbo Ke, Kaiwen Tian, Jianru Nie, Shaoming Huang, Xiaosong Song, Zhiyong Xian

**Affiliations:** 1 Department of Urology, Guangdong Provincial People’s Hospital’s Nanhai Hospital, Foshan, China; 2 Department of Urology, Guangdong Provincial People’s Hospital (Guangdong Academy of Medical Sciences), Southern Medical University, Guangzhou, China; 3 Department of Urology, Ganzhou Hospital of Guangdong Provincial People’s Hospital, Ganzhou Municipal Hospital, Ganzhou, China; 4 Present Address: Department of Urology, Guangdong Provincial People’s Hospital’s Nanhai Hospital, Foshan, China

**Keywords:** BID, Clear cell renal cell carcinoma, Prognosis, Tumor microenvironment

## Abstract

**Background:**

Studies have found that BH3 interacting domain death agonist (BID) is closely related to the occurrence and development of many kinds of tumors. However, little attention has been paid to the situation of BID in clear cell renal cell carcinoma (ccRCC). So, our aim was to explore the effect of BID in ccRCC.

**Methods:**

Survival analysis, ROC curve, correlation analysis and Cox regression analysis were executed to analyze the prognostic value and clinical correlation of BID in ccRCC. The risk prognosis model was constructed in the training cohort and further validated in the internal testing cohort, ICGC cohort, and GEO cohort. Transcriptome sequencing and immunohistochemical staining of clinical specimens were used to validate the results of bioinformatics analysis. The GSEA, ESTIMATE algorithm, CIBERSORT algorithm, ssGSEA, TIDE score, correlation and difference analysis were used to analyze the effects of BID on immune infiltration in tumor microenvironment (TME).

**Results:**

BID was highly expressed in ccRCC tissues, which was verified by transcriptome sequencing and immunohistochemical staining of clinical specimens. Patients with high expression of BID had a worse prognosis. BID is an independent prognostic factor for ccRCC. The prognostic model based on BID can accurately predict the prognosis of patients in different cohorts. In addition, the expression levels of BID was closely related to immunomodulatory molecules such as PD-1, LAG3, and CTLA4. Enrichment analysis indicated that BID was significantly enriched in immune-related responses and cancer-related pathways. The change of BID expression mediates different characteristics of immune infiltration in TME.

**Conclusions:**

BID is highly expressed in ccRCC, which is a reliable biomarker of ccRCC prognosis. It is closely related to TME, and may be a potential target for immunotherapy in patients with ccRCC.

## Introduction

Renal cell carcinoma (RCC) is one of the ten most commonly diagnosed cancers worldwide, accounting for approximately 3% of adult cancers [1]. Every year, the number of new cases and deaths of RCC in the world is gradually increasing [2]. Clear cell renal cell carcinoma (ccRCC) is the most common and fatal histological subtype, exceeding 80% of RCC [3]. Surgical resection is the first choice for early ccRCC, while chemotherapy, targeted drugs and immunotherapy are the main treatment for advanced or/and metastatic ccRCC [4]. Unlike other urological tumors, ccRCC is insensitive to traditional chemotherapy and radiotherapy [5]. Although significant progress has been made in targeted therapy and immunotherapy, the 5-year survival rate of advanced or metastatic ccRCC is still unsatisfactory [6], and the low response rate of immunotherapy also limits its therapeutic effect [7]. Considering the limitations of ccRCC treatment and poor prognosis, we urgently need to explore potential therapeutic targets to improve the prognosis of ccRCC. Identifying new biomarkers and exploring risk prediction models to optimize treatment strategies remains an important work at present.

Increasing evidence shows that the tumor microenvironment (TME) is widely involved in tumor development and prognosis [8], which plays a dominating role in tumorigenesis, immune escape, progression and metastasis [9]. TME is composed of cellular components, extracellular matrix and interstitial fluid. The cellular components of the TME include tumor cells, stromal cells, endothelial cells of blood vessels and lymphatics, neuronal cells, and infiltrating immune cells [10]. Among them, immune cells are the main cellular components, and they drive or prevent tumor progression by participating in various immune responses and activities [11]. Unlike other types of tumors, RCC has significant infiltration of immune cells, including T cells, natural killer cells and macrophages [12]. ccRCC has the highest infiltration degree of T cell in all tumors [13, 14]. However, highly infiltrated CD8 + T cells may be associated with a poor prognosis of ccRCC [15]. Further exploration showed that most of these CD8 + T cells were depleted and functionally deficient, accompanied by high expression of immune checkpoints [16–18]. This shows that the microenvironment of ccRCC has a very complex regulation mechanism, and the tumor immune microenvironment may be a key factor affecting the response of immunotherapy. A number of evidence also shows that ccRCC has high immunogenicity [19], indicating the existence of tumor preheterogeneity in patients, resulting in the low responsiveness to immunotherapy, which highlights the key role of TME in the occurrence and progression of ccRCC. Therefore, exploring the effect of genes on TME and the characteristics of immune infiltration may provide a direction for understanding the potential mechanism of the occurrence and development of ccRCC and developing more effective immunotherapy strategies.

BH3 interacting domain death agonist (BID), a pro-apoptotic member of the BH3-only Bcl-2 family, is cleaved into an active form of tBID by caspase-8 in response to the connection of death receptors [20], which in turn induces apoptosis [21]. BID protein not only participates in external apoptosis signal transduction [22], but also mediates DNA damage response [23]. As an immune gene, BID is closely correlated with the occurrence and development of many tumors. It was reported that the deletion of BID can inhibit cell carcinogenesis in liver, although this genetic change can promote tumorigenesis in myeloid cells [24]. The depletion of hepatocyte-specific BID can reduce tumor development by inhibiting inflammation-associated compensatory proliferation [25]. In addition, BID can enhance the response of ovarian cancer cells to cisplatin and enhance the antitumor effect [26]. Recently, it has been found that BID may be associated with the progression of esophageal squamous cell carcinoma [27]. However, the research on the effect of BID on tumor biology is not deep enough, and its clinical prognostic value for ccRCC is still unclear. In addition, the role of BID in ccRCC and its impact on TME also need to be further explored.

Therefore, the purpose of this study is to explore the expression levels of BID in ccRCC, analyze its prognostic value, and provide help for risk assessment and survival prediction of patients. And explore its effects on TME through a variety of methods to explore the potential mechanism of BID in the occurrence and progression of ccRCC, and provide a theoretical basis for the development of new immunotherapy targets.

## Materials and methods

### Data acquisition and collation

Transcriptome data and clinical information of 539 ccRCC tumor samples and 72 matched normal kidney tissue samples were obtained from the the Cancer Genome Atlas (TCGA) database (https://portal.gdc.cancer.gov/). The progress free survival (PFS) of ccRCC patients was obtained from the UCSC Xena database (https://xenabrowser.net/). The somatic mutation information and tumor mutation burden (TMB) of ccRCC patients were also obtained from the TCGA database. Excluding the samples without complete clinical parameters and survival time of less than 90 days, the remaining 457 cases were randomly divided into training cohort and internal testing cohort at a ratio of 7:3. In addition, we obtained gene transcriptome information and clinical information of 80 eligible ccRCC tumor samples from the International Cancer Genome Collaboration (ICGC) database, as the ICGC external cohort. Three ccRCC datasets (GSE29609、GSE40435、GSE53757) were obtained from the Gene Expression Omnibus (GEO) database (https://www.ncbi.nlm.nih.gov/geo/). Among them, the GSE29609 dataset contains gene transcriptome information and clinical information of 39 cases of ccRCC, as the GEO external cohort. Then, GSE40435 dataset and GSE53757 dataset were used to validate the differential expression analysis of BID in the TCGA database.

### Differential expression of BID in ccRCC and normal kidney tissue

We extracted the mRNA levels of BID in ccRCC and normal kidney tissues from TCGA database, GSE53757 dataset and GSE40435 dataset and corrected them for mutual comparison. The expression difference of BID between ccRCC and normal kidney tissues was analyzed by executing the "ggpubr" R package in the TCGA dataset. GSE53757 dataset and GSE40435 dataset were used to validate the analysis results of the TCGA dataset.

### The predictive value and clinical relevance of BID for ccRCC

Patients were divided into BID low- and high-expression group by the median of BID expression in the TCGA dataset. The Kaplan–Meier survival curves were drew and the difference of overall survival (OS) and PFS were compared between the two groups. In order to evaluate the value of BID in predicting the prognosis of ccRCC patients, we judged the reliability by the receiver operating characteristic (ROC) curve and the area under the curve (AUC). In addition, Wilcoxon rank sum test was executed to investigate the relevance between the expression levels of BID and the clinical characteristics.

### Independent prognostic value of BID for ccRCC

The expression of BID was considered as a prognostic factor, and an univariate Cox prognostic analysis was performed on BID, age, gender, T stage, M stage, histological grade and AJCC clinical stage to evaluate the effect of various parameters on the OS of patients. In order to exclude the influence of other confounding factors, we then conducted a multivariate Cox prognostic analysis to determine whether BID can be independent of other clinicopathological features, and to judge whether BID is an independent prognostic factor for ccRCC.

### Construction and verification of BID-related prognostic model

After identifying prognostic factors with independent prognostic value for ccRCC in the above analysis, we constructed a risk prognostic model in the TCGA training cohort via multivariate Cox regression analysis.

$$\mathrm{Risk score }=\sum ({\text{Xi}}\times {\text{Yi}})$$

where Xi and Yi represent the risk regression coefficient of each parameter and the value of the variable, respectively. Low- and high-risk group were distinguished via the median value of patients’ risk score. We plotted the Kaplan–Meier survival curves of patients to investigate the difference in prognosis between the two groups based on the "survival" and "survminer" R packages. The performance of the model was evaluated by the AUC values of the ROC curves. Subsequently, we drew the patient's risk distribution curve and survival status map through the "pheatmap" R package to further research the relationship between the risk score and the prognosis of patients. In addition, principal component analysis (PCA) was run to determine whether the model could well identify patients with different risks. We also established multi-index ROC curves to judge whether the risk score based on this model is better than general clinicopathological information. Finally, same analysis were conducted in the TCGA internal testing cohort, ICGC external cohort and GEO external cohort to further verify the model.

### Establishing a nomogram to predict the prognosis of ccRCC patients

In order to predict the prognosis of ccRCC patients more effectively and promote the clinical application of the model, we combined the mRNA levels of BID and the clinical characteristics with independent prognostic value to construct a nomogram. The calibration curves were used to visualize the performance of the nomogram to assess the difference between predicted results and actual observations, with the 45°slope representing the best predictive performance.

### Gene set enrichment analysis (GSEA)

The GSEA analysis was implemented by the GSEA software with 1000 cycles of genome sequencing per analysis. The Hallmark pathways obtained from the MSigDB database (http://www.broad.mit.edu/gsea/msigdb/) were used for functional annotation of the above enrichment analysis. When the absolute value of the normalized enrichment score (NES) is ≥ *1.0*, the NOM *P* value is < *0.05*, and the FDR q-value is < *0.25*, it can be considered as a meaningfully enriched gene set.

### Tumor microenvironment and immune cell infiltration

We calculated the TME score (immune score, stromal score, ESTIMATE score) of ccRCC patients by the ESTIMATE algorithm, and compared the differences between BID high- and low-expression group to further evaluate the impact of BID on the TME. Subsequently, we performed a Spearman correlation test to study the correlation between the expression of BID and TME. We evaluated the infiltration fractions of 22 types of immune cells in ccRCC patients by the CIBERSORT algorithm. Spearman correlation test was used to further explore the correlation between the expression of BID and the infiltration levels of immune cells in the microenvironment. Then, the differential analysis of the immune cell infiltration between the BID low- and high-expression group was performed, and the difference in the abundance of all cells was displayed on the radar map generated by the "fmsb" R package. Finally, we performed a prognostic analysis of differentially infiltrating immune cells to research the relevance between the changes of immune cell content and the prognosis of ccRCC patients.

### Single sample gene set enrichment analysis (ssGSEA)

In order to further explore the effect of BID on tumor immune microenvironment, we evaluated the infiltration scores of 16 immune cells and the activity of 13 immune-related pathways in ccRCC patients by performing ssGSEA based on "GSVA" R package and "GSEABase" R package. Then, the differences between the BID high- and low-expression group were compared.

### Correlation and difference analysis of immune checkpoints

Dysregulation of immune checkpoint expression is overtly related with drug resistance and immune escape. So, we performed Spearman correlation analysis between BID and 10 common immune checkpoints to study their correlation. In addition, we also performed differential expression analysis of these immune checkpoints between BID high- and low-expression group to further evaluate the potential impact of BID on them.

### Tumor mutation burden and somatic mutations

First, a Spearman correlation analysis was conducted on the TMB and the expression levels of BID to explore their correlation. Then, we performed a differential analysis of TMB in the BID high- and low-expression group to evaluate the difference of TMB in different BID groups. Next, patients were classified into TMB-high and TMB-low group based on the median of TMB, and compared the survival difference between two group by Kaplan–Meier analysis to evaluate the relationship between TMB and the prognosis of ccRCC patients. Finally, we used the 'maftools' R package to generate waterfall plots to show the distribution of somatic mutations in the BID high- and low-expression group.

### Tumor immune dysfunction and exclusion (TIDE) score

The TIDE characteristics calculated from untreated tumor data can predict patients' clinical response to immune checkpoint inhibitors (ICIs) [28]. The TIDE score of ccRCC patients were obtained from the TIDE website (http://tide.dfci.harvard.edu). We performed a differential analysis of these indicators between the BID high- and low-expression group to evaluate the possible impact of BID on ICIs therapy in ccRCC patients.

### Tissue samples and transcriptome sequencing

A total of 18 tumor samples and 6 paracancerous samples were collected from 6 ccRCC patients treated at Guangdong Provincial People's Hospital. Transcriptome sequencing was performed on all samples according to the manufacturer's protocol. The study was conducted in accordance with the Declaration of Helsinki (as revised in 2013). The study was approved by the Ethics Committee of Guangdong Provincial People's Hospital (IRB number: KY-Z-2021-657-01). All patients have signed the relevant informed consent and filed.

### Immunohistochemical staining

We retrieved and downloaded the immunohistochemical staining results of BID in ccRCC and normal kidney tissues from the Human Protein Atlas (HPA) database (https://www.proteinatlas.org/) to initially identify the expression of BID protein in ccRCC tissues and normal kidney tissues. In addition, immunohistochemical experiments were also carried out on tumor samples collected from the hospital to further verify the expression of BID protein. The BID primary antibody was purchased from the Proteintech (10,988-1-AP). The HRP-labeled goat anti-rabbit IgG secondary antibody was obtained from the Servicebio (GB23303). The results of immunohistochemical staining were observed under a microscope by two experienced pathologists. The hematoxylin-stained cell nuclei were blue, and the positive expression of DAB was brownish yellow.

### Statistical analysis

The Wilcoxon rank sum test was used to compare the differences between two groups, and the correlation between variables was assessed by the Spearman correlation test. All statistical analysis methods and R packages were performed in R software (version 4.0.4) and *P* < *0.05* was considered statistically significant.

## Results

### BID is highly expressed in ccRCC tissues

**Fig. 1:**
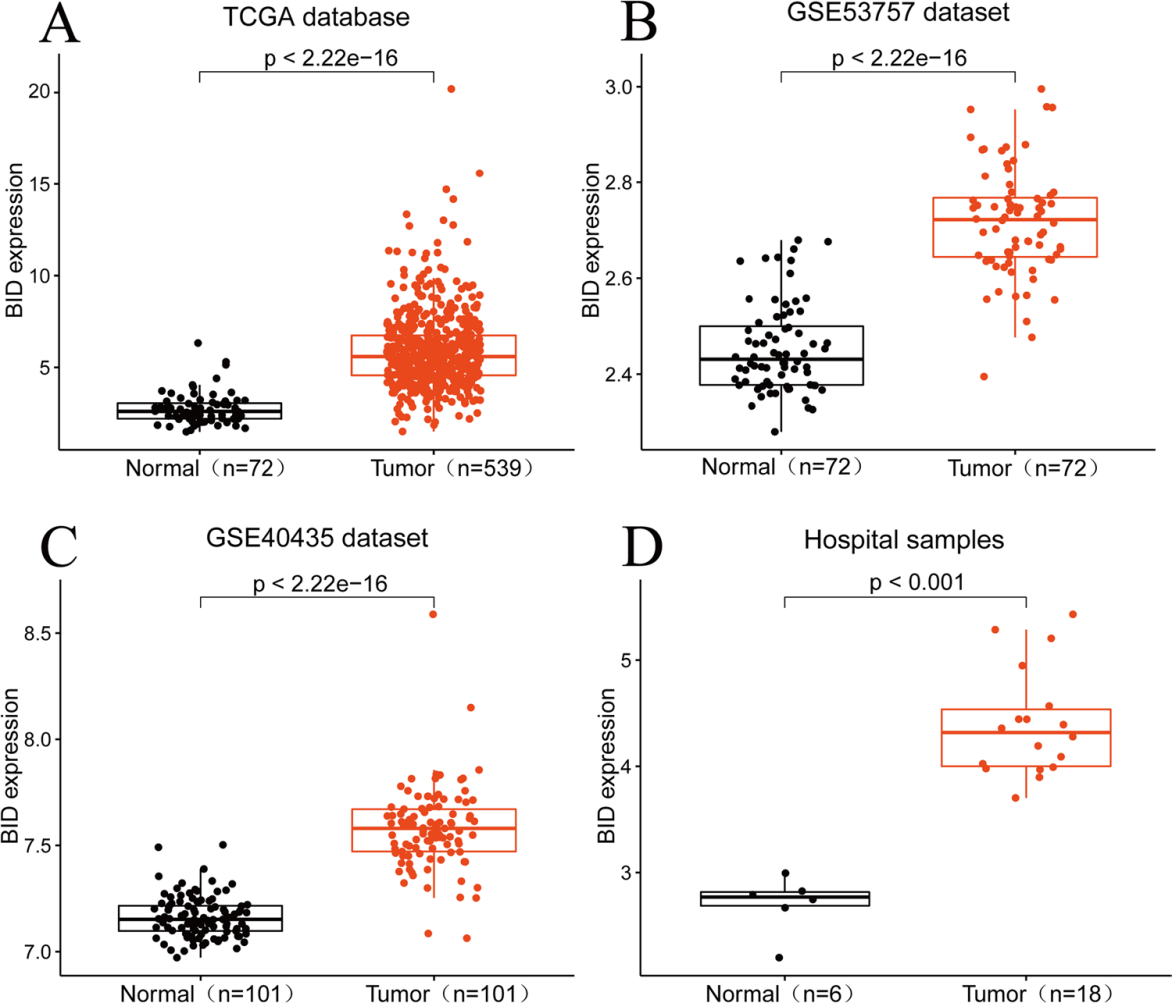
BID is highly expressed in ccRCC tissues. **A** In the TCGA database, the expression levels of BID was significantly up-regulated in ccRCC tissues. **B**, **C** The differential expression of BID in normal kidney tissues and ccRCC tissues in the GEO dataset. **D** The transcriptome sequencing of tumor specimens confirmed the differential expression of BID in normal renal tissues and ccRCC tissues

We used transcriptome information of 539 ccRCC tumor samples and 72 matched paracancerous samples to explore the differential expression of BID. Results as shown in Fig. 1A, compared with normal renal tissue, the expression levels of BID in ccRCC tissue was significantly up-regulated (*P* < *0.001*). In addition, we validate the result by the GSE53757 dataset and GSE40435 dataset of the GEO database. Consistent with our expected result, the differential expression of BID was further verified in tumor tissues and normal renal tissues (Fig. 1B, C, P < *0.001*). Moreover, the results of transcriptome sequencing of tumor samples also demonstrated that BID was highly expressed in ccRCC tissues and lowly expressed in normal kidney tissues (Fig. 1D, P < *0.001*).

### The prognostic value of BID in ccRCC

**Fig. 2:**
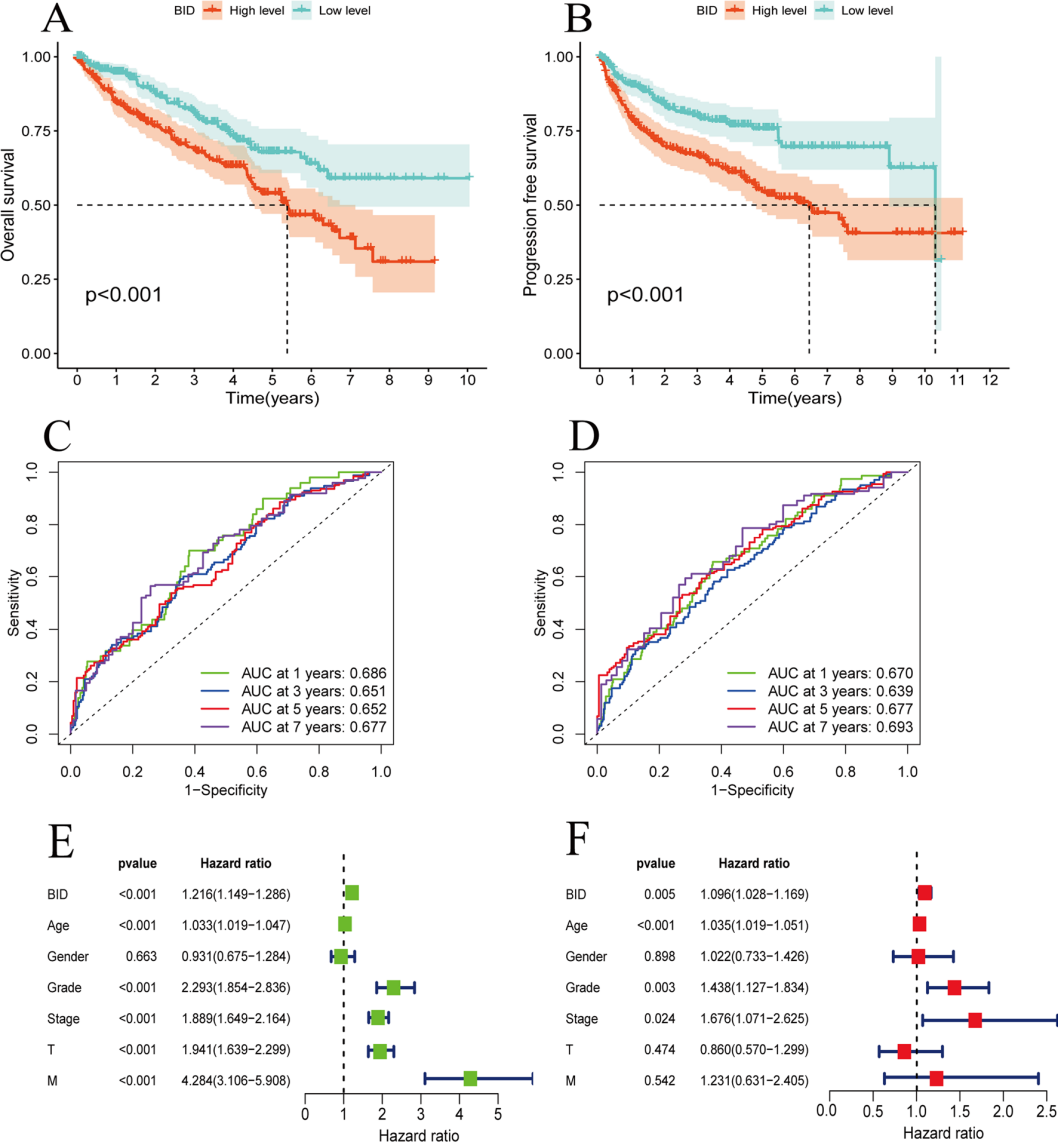
The prognostic value of BID in ccRCC. **A** The Kaplan–Meier survival curve of BID predicting the OS of patients. **B** The Kaplan–Meier survival curve of BID predicting the PFS of patients. **C** The ROC curve of BID predicting the OS of 1-year, 3-year, 5-year and 7-year for ccRCC patients. **D** The ROC curve of BID predicting the PFS of 1-year, 3-year, 5-year and 7-year for ccRCC patients. **E** The result of univariate Cox independent prognostic analysis. **F** The result of multivariate Cox independent prognostic analysis

Kaplan–Meier survival curves revealed a shorter OS and PFS for BID high expression group patients compare to patients in BID low expression group (Fig. 2A, B, P < *0.001*). We introduced ROC curves to evaluate the predictive potential of BID on the prognosis of ccRCC patients. The results revealed that the AUC values of the ROC curves of BID predicting the 1-, 3-, 5- and 7-year OS of patients were 0.686, 0.651, 0.652 and 0.677, respectively (Fig. 2C). The AUC values of the ROC curves of BID predicting the 1-, 3-, 5- and 7-year PFS of patients were 0.670, 0.639, 0.677 and 0.693, respectively (Fig. 2D). This suggests that BID has good prognostic predictive ability for ccRCC patients. The results of univariate Cox independent prognostic analysis demonstrated that BID (HR = 1.216, *P* < *0.001*), age (HR = 1.033, *P* < *0.001*), T stage (HR = 1.941, *P* < *0.001*), M stage (HR = 4.284, *P* < *0.001*), histological grade (HR = 2.293, *P* < *0.001*) and clinical stage (HR = 1.889, *P* < *0.001*) were all correlated with the OS of patients (Fig. 2E). After excluding confounding factors by multivariate Cox prognostic analysis, BID was still an independent risk factor for the prognosis of ccRCC patients (Fig. 2F). This indicates BID may be a risk gene of ccRCC and participate in tumor progression.

### Clinical correlation between the expression levels of BID and ccRCC

**Fig. 3:**
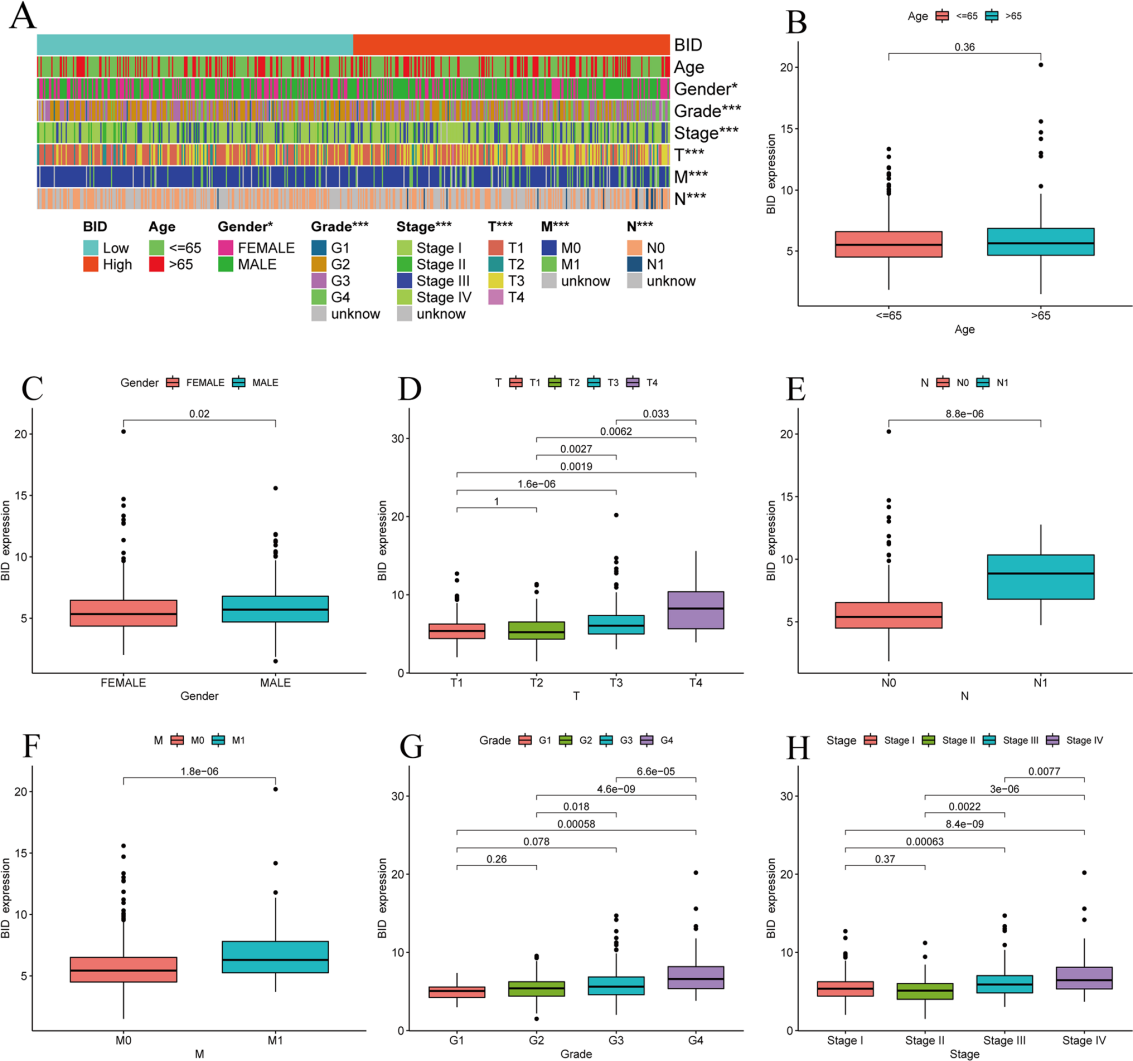
The relationship between the BID and the clinicopathological features of ccRCC patients. **A** The heat map of the correlation. **B** The expression level of BID in different age groups of patients. **C** The expression level of BID in male and female. **D**–**H** The relationship between the expression levels of BID and the T stage, N stage, M stage, histological grade and clinical stage of patients (*: *P* < 0.05; **: *P* < 0.01; ***: *P* < 0.001)

We found BID was clearly associated with common clinicopathological features of ccRCC patients except for age (Fig. 3A). Specifically, there was no difference in the expression levels of BID among different age groups (Fig. 3B), but it was higher in male patients (Fig. 3C). In addition, the high expression of BID may predict worse T stage, N stage, histological grade and clinicopathological stage, and lead to distant metastasis (Fig. 3D–H).

### Construction of BID-related prognostic model

In the above analysis, we identified the risk factors with independent prognostic value for ccRCC. We constructed a risk prognosis model by multivariate Cox regression analysis in the TCGA training cohort.

$$\mathrm{Risk\, score }=\left(0.023060099\times {\text{age}}\right)+\left(0.088244672\times \mathrm{BID mRNA}\right)+\left(0.306695441\times {\text{grade}}\right)+\left(0.545605079\times {\text{stage}}\right)$$

**Fig. 4:**
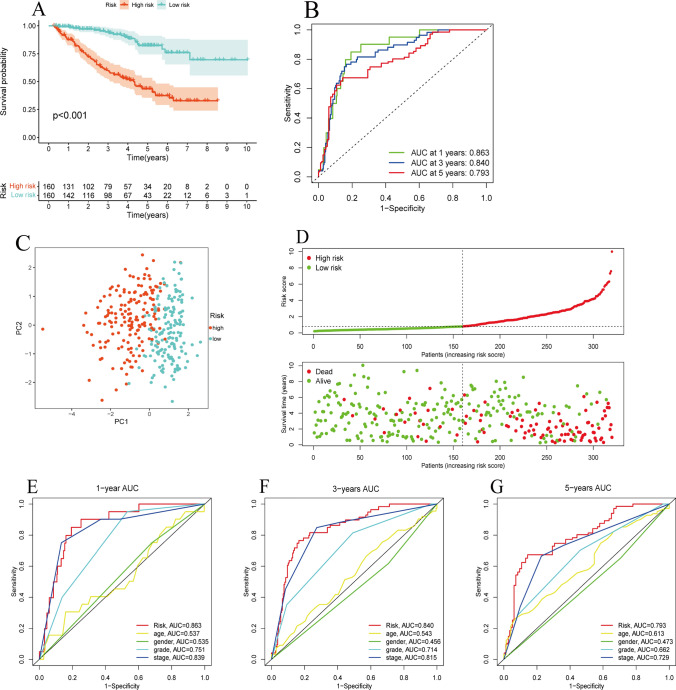
Construction of a BID-related prognostic model. **A** There is a significant difference in OS between the high- and low-risk group. **B** The ROC curve of the model predicting the 1-year, 3-year, and 5-year OS of patients. **C** The result of PCA analysis. **D** The risk distribution curve and survival status plots of patients. **E**–**G** The multi-index ROC curves

The Kaplan–Meier survival analysis demonstrated a distinctly decreased OS of patients with high riskscore (Fig. 4A, P < *0.001*). The AUC values of the ROC curves for predicting the 1-, 3-, and 5-year OS via the model were 0.863, 0.840, and 0.793, respectively, demonstrating the good predictive performance of the model (Fig. 4B). In addition, the results of PCA showed that the model could accurately distinguish patients with different risks (Fig. 4C). As the risk score increased, the patient's survival time decreased and the number of deaths significantly increased, indicating the risk score was negatively correlated with the prognosis of patients (Fig. 4D). Moreover, the multi-index ROC curves revealed that the AUC value of the model was better than that of any single clinical trait, no matter at 1 year, 3 years, or 5 years, showing good prognostic predictive ability (Fig. 4E–G).

### Validation of BID-related prognostic model

**Fig. 5:**
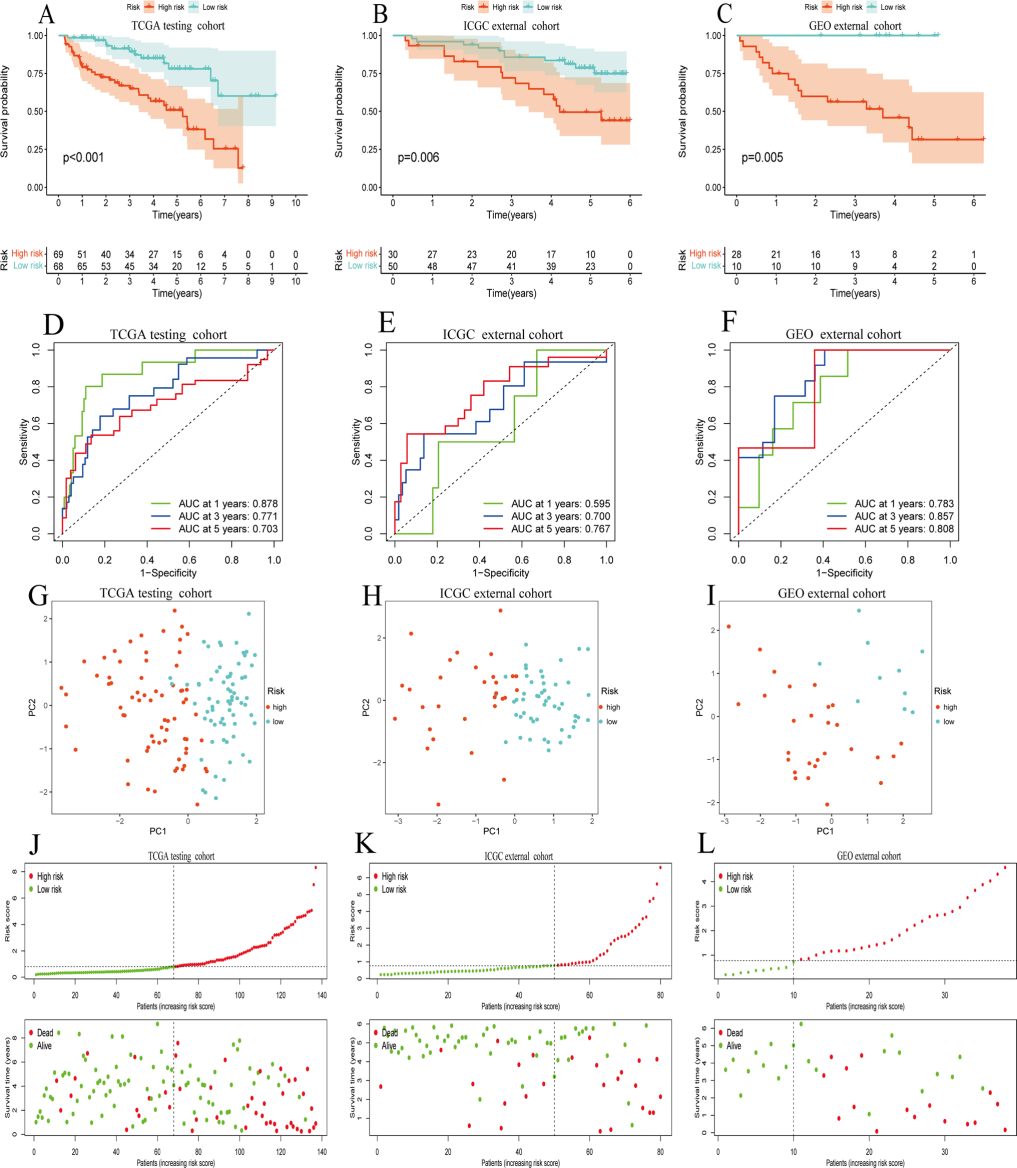
Internal and external validation of BID-related prognostic models. **A**–**C** Kaplan–Meier survival curves of patients in the high- and low-risk groups in the testing cohort, ICGC external cohort and GEO external cohort. **D**–**F** The ROC curves of the prognostic model in the testing cohort, ICGC external cohort and GEO external cohort. **G**–**I** The results of the PCA analysis for the testing cohort, ICGC external cohort and GEO external cohort. **J**–**L** The risk distribution curve and survival status plots for the testing cohort, ICGC external cohort and GEO external cohort

We validated the model’s accuracy and effectiveness in the testing cohort, ICGC external cohort and GEO external cohort. Kaplan–Meier survival analysis showed a shorter OS for patients in the high-risk group in the testing cohort (Fig. 5A, P < *0.001*). Interestingly, consistent with the result of the TCGA training cohort, the same difference in OS was observed between two groups in ICGC external cohort and GEO external cohort (Fig. 5B, C, P < *0.001*). In the testing cohort, the AUC values of 1 -, 3-and 5-year OS of the prognostic model were 0.878, 0.771 and 0.703, respectively (Fig. 5D). In the ICGC external cohort, the AUC values of 1 -, 3-and 5-year OS of the prognostic model were 0.595, 0.700 and 0.767, respectively (Fig. 5E). In the GEO external cohort, the AUC values of 1 -, 3-and 5-year OS of the prognostic model were 0.783, 0.857 and 0.808, respectively (Fig. 5F). In addition, the results of the PCA of the three cohorts showed that the model could also accurately distinguish patients with different risks (Fig. 5G–I). The risk distribution curve and survival status map of the three cohorts also demonstrated that the risk score was negatively correlated with the prognosis of patients (Fig. 5J–L). Anyway, these results revealed the accuracy and effectiveness of the BID-related risk model in predicting the prognosis of ccRCC patients.

### A nomogram for predicting the prognosis of ccRCC

**Fig. 6:**
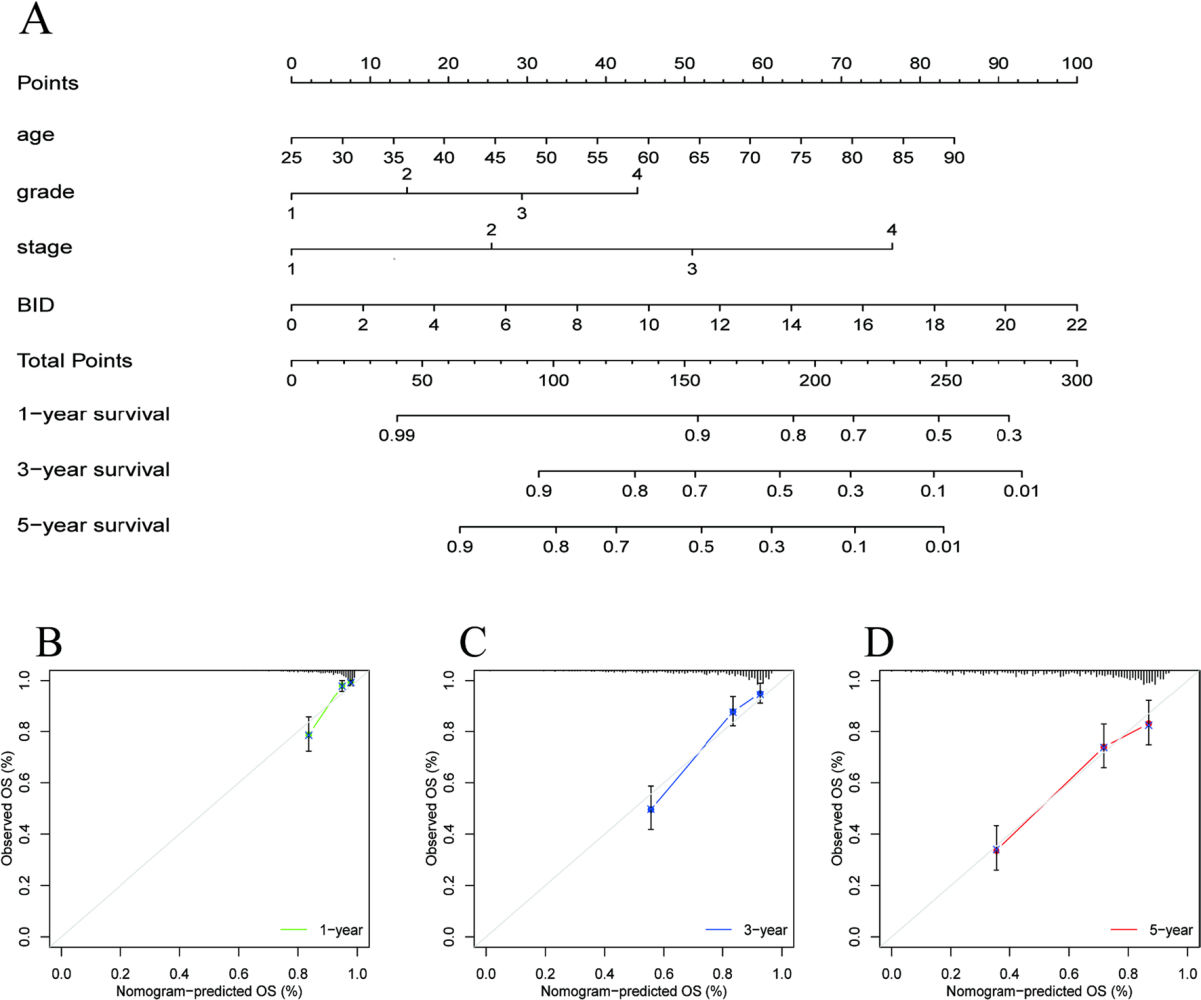
The Nomogram for predicting prognosis of ccRCC. **A** Nomogram that can predict 1-, 3-, and 5-year survival rates of ccRCC. **B**–**D** The calibration curve of the nomogram demonstrated good agreement between the actual survival rate and predicted survival rate

We constructed a nomogram that can predict the 1 -, 3 -, and 5-year OS for ccRCC. (Fig. 6A). It combines the expression levels of BID and three clinical characteristics with independent prognostic (age, histological grade, clinical stage), which can be better used for clinical assessment of individual survival probability. The calibration curve of the nomogram demonstrated good agreement between the actual survival rate of the patients and the predicted survival rate (Fig. 6B–D), revealing the superior predictive performance of the nomogram.

### The GSEA of BID

**Fig. 7:**
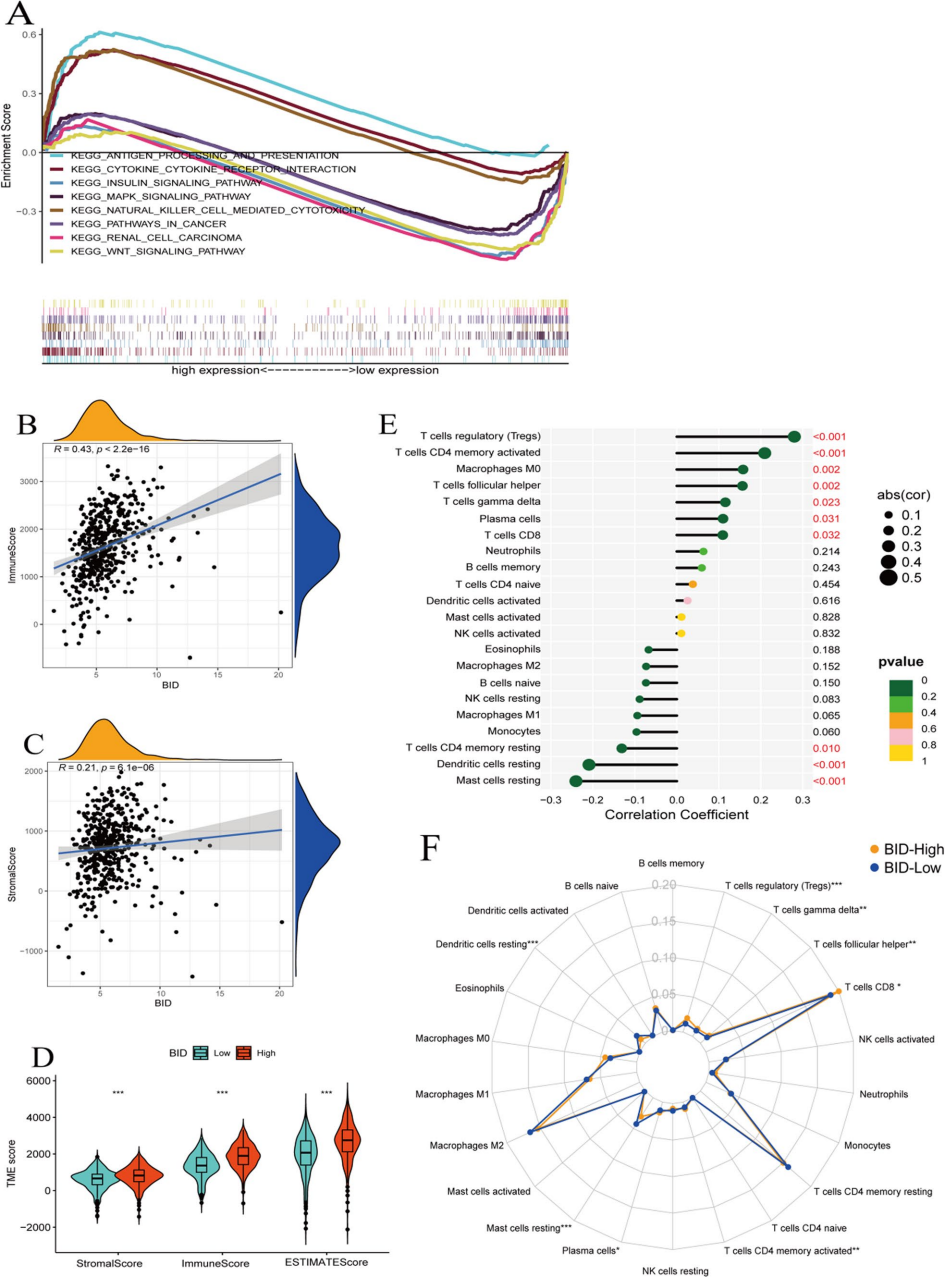
BID-related enrichment pathways and characteristics of immune infiltration. **A** The GSEA of BID. **B** Correlation between the expression of BID and the immune score of ccRCC samples. **C** Correlation between the expression of BID and the stromal score of ccRCC samples. **D** Differences in TME scores of ccRCC samples between BID low- and high-expression group. **E** The correlation between the expression levels of BID and the immune cells in TME. (F) Differences in infiltration of immune cells between BID high- and low-expression group (*: *P* < 0.05; **: *P* < 0.01; ***: *P* < 0.001)

To research the potential mechanism of BID in the occurrence and development of ccRCC, we performed GSEA on the BID. The results manifested that BID overexpression was distinctly enriched in immune processes such as cytokine_cytokine_receptor_interaction, natural killer cell-mediated cytotoxicity, and antigen processing and presentation; while the down-regulation of BID was significantly enriched in insulin signaling pathway, WNT signaling pathway, renal cell carcinoma, pathways in cancer and MAPK signaling pathway (Fig. 7A).

### BID-mediated tumor immune infiltration features

Accumulating evidence indicates that TME plays a central role in tumor initiation, progression and metastasis. The results of correlation analysis showed that the correlation coefficient between the expression of BID and immune score was 0.43 (Fig. 7B), and that between BID and stromal score was 0.21 (Fig. 7C), which indicates that the expression levels of BID was positively correlated with TME score in patients with ccRCC. Besides, the samples with high expression of BID had higher immune score, stromal score and ESTIMATE score (Fig. 7D , P < *0.001*), indicating that the tumor tissue with high expression of BID may have higher content of immune cells and stromal cells, and higher tumor purity.

**Fig. 8:**
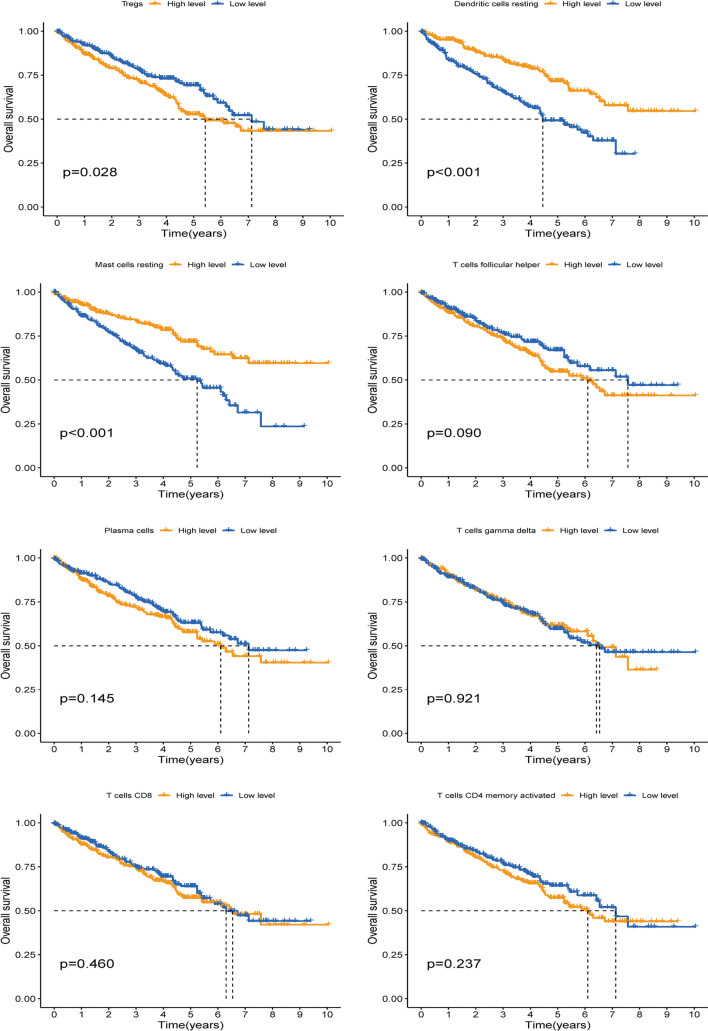
Survival analysis of of 8 kinds of immune infiltration cells

The infiltration of immune cells in TME is complex and diverse, which is intimately correlated to the efficacy of immunotherapy. The CIBERSORT analysis results demonstrated that the expression levels of BID was correlated with 10 kinds of immune cells in the TME (Fig. 7E). Specifically, the expression levels of BID was positively correlated with the infiltration levels of T cells regulatory (Tregs), T cells CD4 memory activated, Macrophages M0, T cells follicular helper, T cells gamma delta, plasma cells and T cells CD8; while that was negatively correlated with the infiltration levels of Mast cells resting, Dendritic cells resting and T cells CD4 memory resting. The radar map showed that the infiltration levels of Tregs, T cells gamma delta, T cells follicular helper, T cells CD8, T cells CD4 memory activated and plasma cells were higher in the BID high expression group, while the infiltration levels of Mast cells resting and Dendritic cells resting were decreased (Fig. 7F). Survival analysis of these differentially infiltrated immune cells revealed that high infiltration of Tregs and low infiltration of Mast cells resting and Dendritic cells resting were associated with poor prognosis, while the infiltration levels of T cells CD8, T cells gamma delta, T cells follicular helper, T cells CD4 memory activated and plasma cells had on correlation with prognosis of patients (Fig. 8).

### ssGSEA

**Fig. 9:**
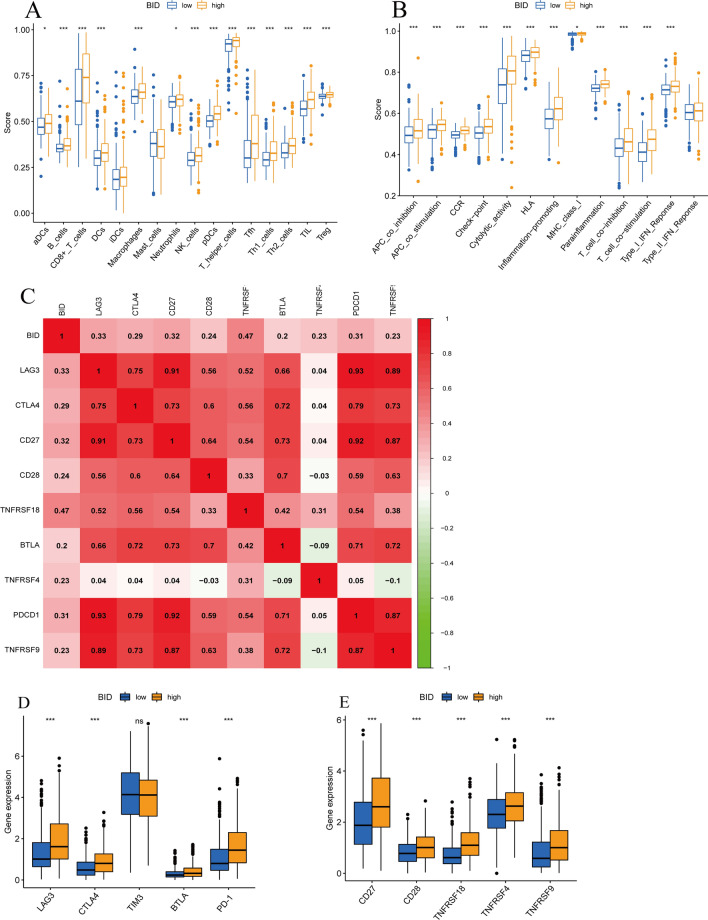
The results of ssGSEA for BID and the relationship between BID and common immune checkpoints. **A** The differences in enrichment fractions of 16 types of immune cells between BID high- and low-expression group. **B** The difference of the activity of 13 immune-related pathways between BID high- and low-expression group. **C** Correlation between BID and 9 immune checkpoints. **D**, **E** The expression levels of 10 immune checkpoints in BID high- and low-expression group (*: *P* < *0.05*; ***: *P* < *0.001*)

The ssGSEA can evaluate the score and immune activity of immune infiltrating cells in tumor samples, so as to further explore the effect of BID on tumor immune microenvironment. The results of ssGSEA showed that the samples with high expression of BID usually had higher immune cell infiltration scores, and among the comparison of 16 types of immune cells, the infiltration scores of 14 types of immune cells were increased (Fig. 9A). In addition, the activity of immune-related pathways in samples with BID high expression was also higher. Except for the Type_II_IFN_Reponse, the activities of 12 immune pathways in samples with BID high expression were higher than those in samples with BID low expression (Fig. 9B).

### The relationship between BID and immune checkpoints

We evaluated the relativity between BID and common immune checkpoints. The results showed that 4 immunostimulatory molecules and 5 immunosuppressive molecules were significantly correlated with the expression of BID (Fig. 9C). Then, differential expression analysis was performed on these immune checkpoints between BID high- and low-expression group. We found that these 9 immune checkpoint genes were significantly up-regulated in samples with BID high expression, while down-regulated in the samples with BID low-expression (Fig. 9D, E, P  < *0.001*).

### Tumor mutation burden and somatic mutations

**Fig. 10:**
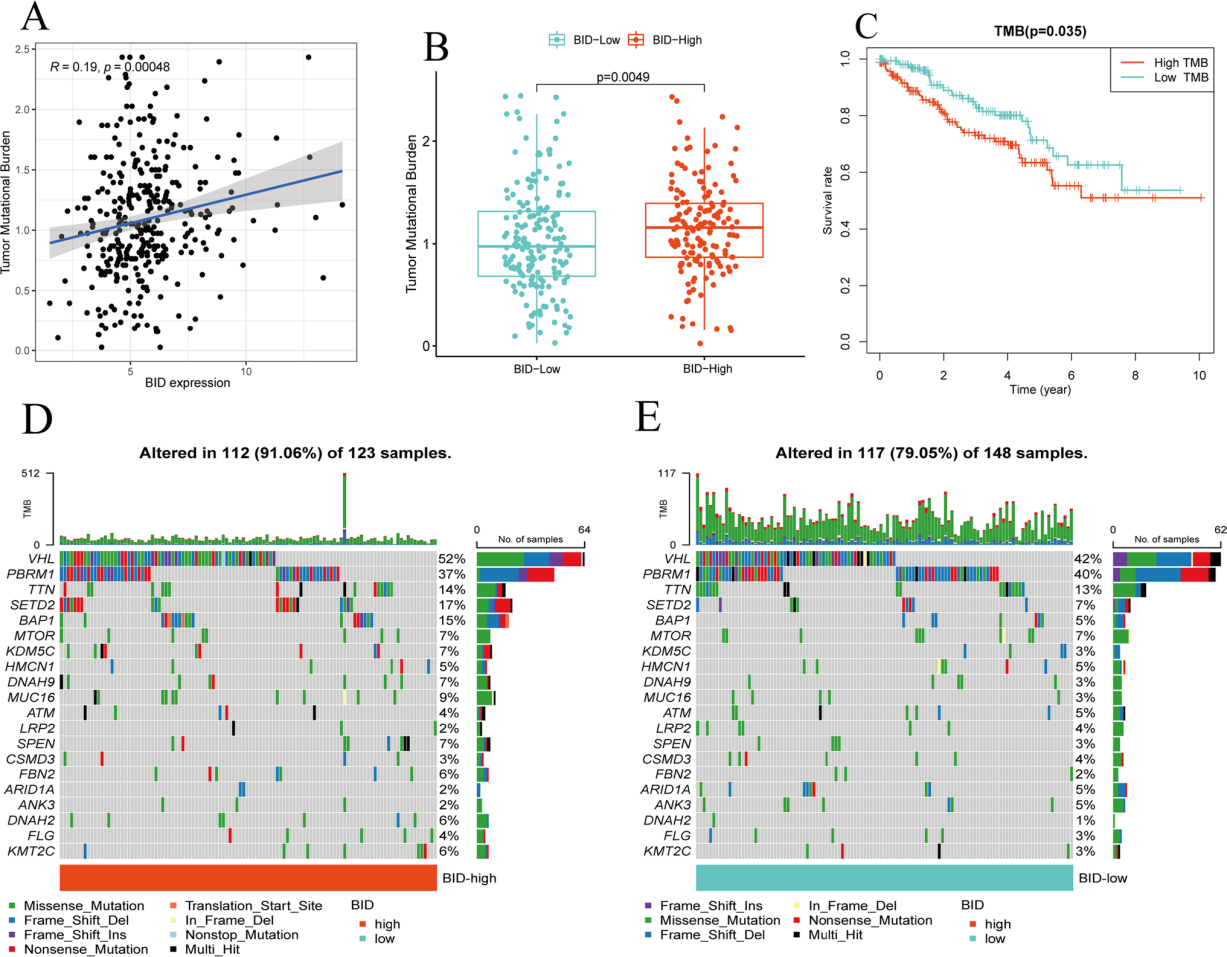
The relationship of BID to TMB and somatic mutations. **A** Correlation between BID and TMB of ccRCC samples. **B** The difference in TMB between BID high- and low-expression group. **C** The survival analysis of TMB. **D** Distribution of somatic mutations in samples with BID high expression. **E** Distribution of somatic mutations in samples with BID low expression

TMB is a key factor affecting tumor immune response and immunotherapy. We found that the expression levels of BID was positively correlated with TMB in ccRCC samples (Fig. 10A). The results of differential analysis showed that ccRCC samples with BID high expression had higher TMB compared with samples with BID low expression (Fig. 10B). Kaplan–Meier survival analysis demonstrated that patients in the TMB high group had a decreased OS compared with the TMB low group (Fig. 10C). Then, we used waterfall plot to show the mutation status of the 20 genes with the highest somatic mutation frequency between BID high- and low-expression groups (Fig. 10D, E). We found that missense mutations were the most common type in ccRCC samples, followed by frameshift deletions and nonsense mutations. The VHL mutation and PBRM1 mutation were the most common in both BID high- and low-expression groups. The biggest differences of mutations between groups were VHL, SETD2 and BAP1 mutations. Specifically, VHL mutation, SETD2 mutation and BAP1 mutation were more common in BID high expression group (52% vs. 42%, 17% vs. 7%, 15% vs. 5%).

### TIDE scores

**Fig. 11:**
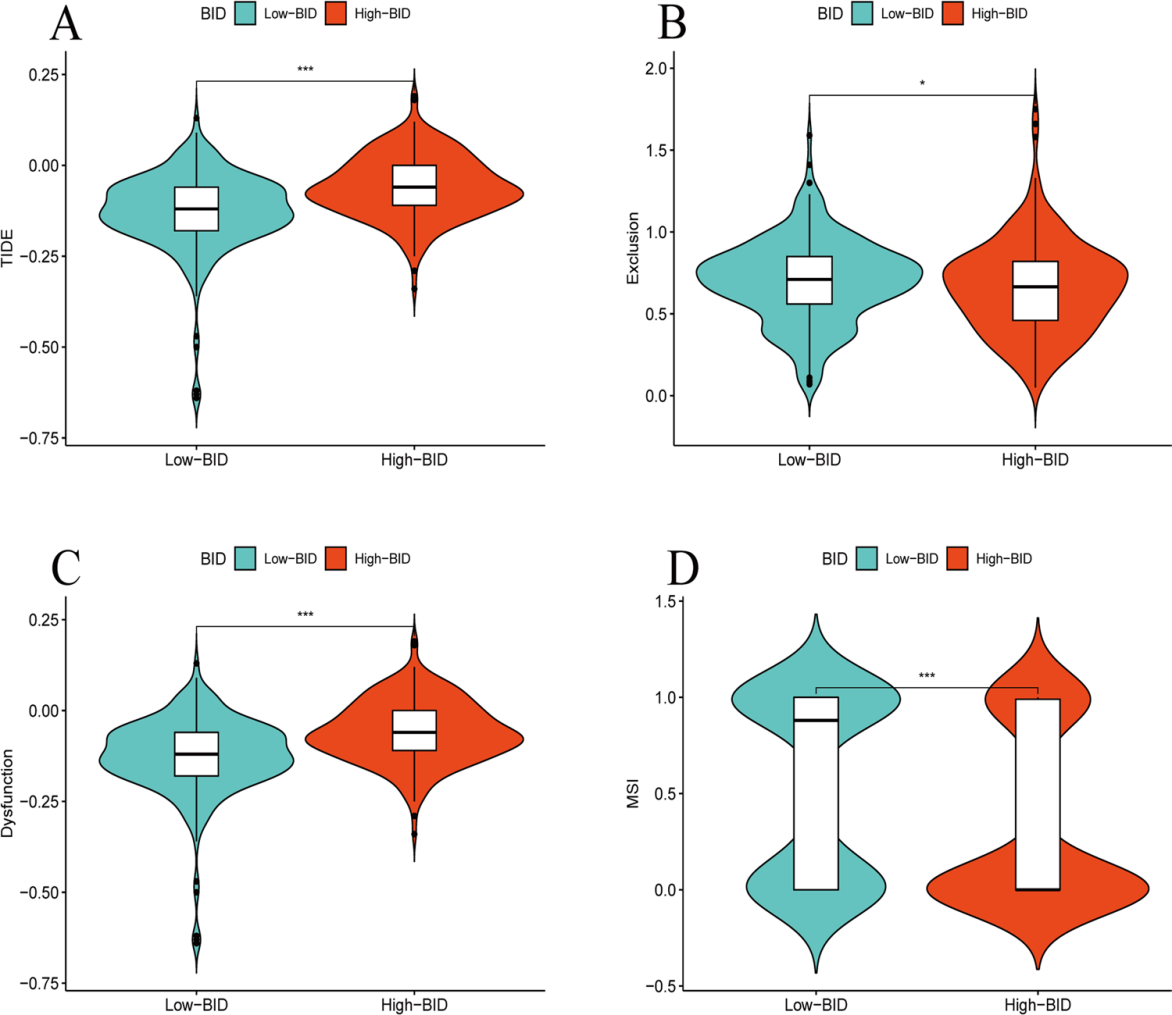
The TIDE scores of ccRCC. **A** Differences in TIDE scores of ccRCC samples between BID high- and low-expression group. **B** Differences in T cell exclusion scores of ccRCC samples between two groups. **C** Differences in T cell dysfunction scores of ccRCC samples between two groups. **D** Differences in MSI of ccRCC samples between two groups (*: *P* < *0.05*; **: *P* < *0.01*; ***: *P* < *0.001*)

The TIDE scores can predict the response of patients to ICIs. We found that the samples with BID high expression had higher TIDE scores (Fig. 11A), suggesting that such patients may benefit less from the treatment of ICIs. The T cell exclusion scores was lower in samples with BID high expression (Fig. 11B), while T cell dysfunction scores was higher (Fig. 11C). Finally, we also compared the differences of MSI between the two groups. The MSI was higher in samples with BID low expression (Fig. 11D).

### The results of immunohistochemical staining

**Fig. 12:**
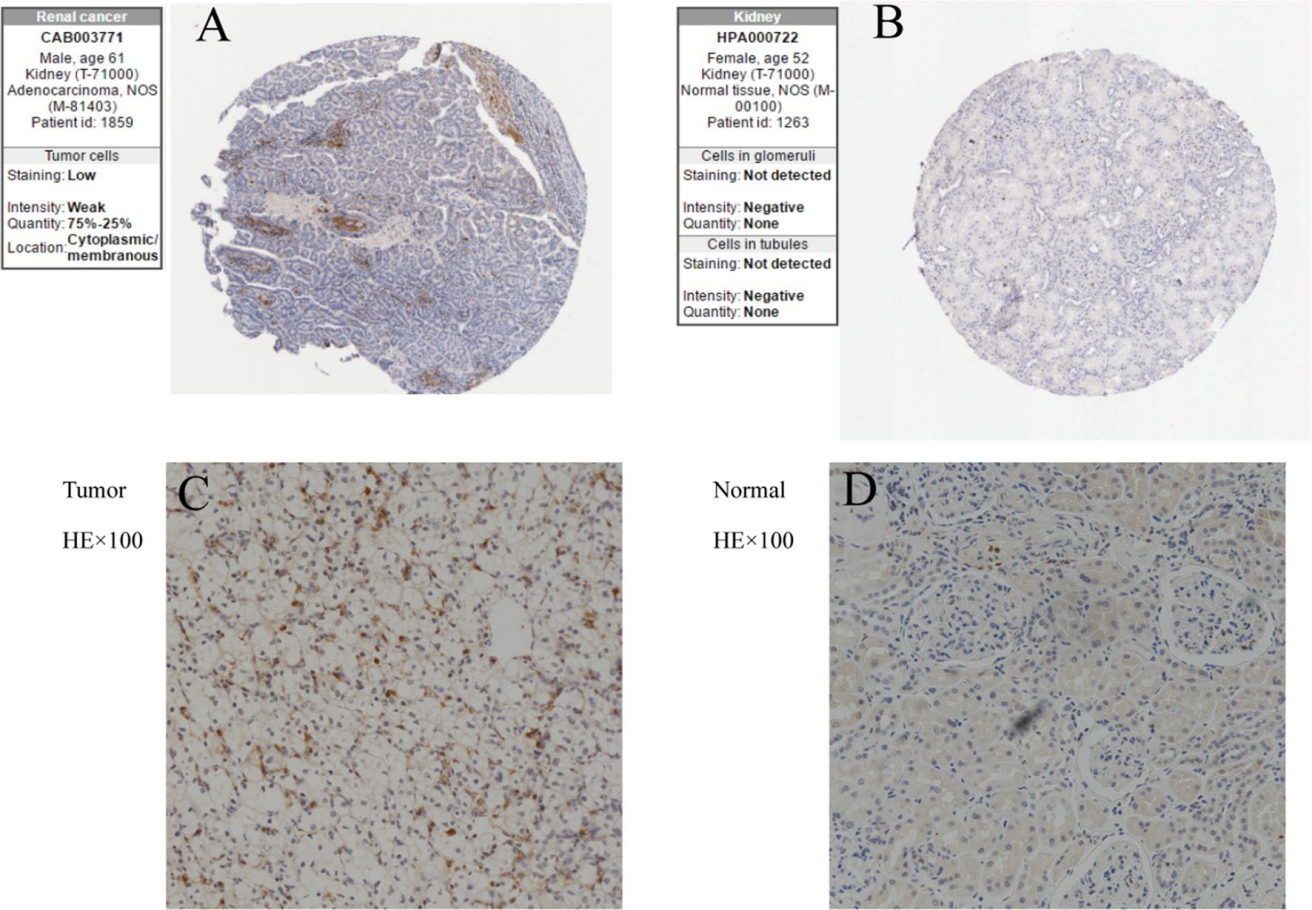
The results of immunohistochemical staining of BID. **A** BID protein was highly expressed in ccRCC tissues in the HPA database. **B** BID protein was lowly or not expressed in normal kidney tissues in the HPA database. **C** BID protein was highly expressed in ccRCC specimens. **D** BID protein was lowly or not expressed in normal kidney specimens

We obtained the results of immunohistochemical staining of BID protein in ccRCC tissue and normal renal tissue from HPA database. From the Fig. 12A, B we can find that BID protein was highly expressed in ccRCC tissues, but low or not expressed in normal renal tissues. The results of immunohistochemical staining of tumor samples collected from the hospital also demonstrated that BID protein was highly expressed in the cytoplasm of ccRCC tissues, while low or no expression in normal renal tissues adjacent to cancer (Fig. 12C, D).

## Discussion

RCC is a relatively common malignant tumor of the genitourinary system with a high mortality rate and the global incidence is on the rise [29, 30]. ccRCC is the most common pathological type and the main cause of death for patients [31]. Growing evidence shows that immunotherapy based on ICIs can achieve anti-tumor effects by stimulating the immune response [32, 33], which can significantly improve the outcome of patients with advanced ccRCC [34, 35]. Unfortunately, only a small number of patients benefit from this treatment, while most patients are ineffective. Moreover, the pathogenesis of ccRCC is still unclear. There is an urgent need to find new biomarkers to predict the prognosis of immune response in patients with ccRCC, in order to promote diagnosis and treatment.

In this study, we found that the expression levels of BID in ccRCC tissues was significantly higher than that in normal renal tissues. This difference was also confirmed by GEO database and transcriptome sequencing of tumor samples. Based on these results, we speculate that BID may play a carcinogenic role through up-regulation of expression in ccRCC, but the specific mechanism remains to be further investigated. The results of clinical correlation analysis and survival analysis demonstrated that the high expression of BID was observably correlated with poor T stage, N stage, M stage, histological grade and clinical stage. However, we also found that the expression levels of BID was no difference among patients with TI-T2, G1-G2, and stage I and II, which suggests that BID may play an vital role in the progression of tumor to advanced stages. Univariate and multivariate Cox regression analysis proved that BID is an independent prognostic factor for ccRCC. In addition, the AUC values of the ROC curve of BID for predicting the OS and PFS of ccRCC patients were very eligible, which indicated that BID has a good predictive ability for the prognosis of patients. These results above suggest that BID may play an essential role in ccRCC progression and can be considered as a eligible prognostic biomarker.

We constructed a risk prognostic model through multivariate Cox regression analysis in the TCGA training cohort. The model has good predictive performance and was further validated in the internal testing cohort, ICGC external cohort and GEO external cohort. Then, we constructed a nomogram that can predict the 1 -, 3-and 5-year OS of patients. It combines the expression levels of BID with three clinical parameters of independent prognosis, which can be better used to evaluate individual survival probability, and further improves the practical value of BID in clinical application.

In order to explore the potential mechanism of BID expression changes in the occurrence and development of ccRCC, we conducted the GSEA to further explore related biological functional pathways. We found that BID high expression was observably enriched in the immune process, while BID down-regulation was enriched in energy metabolism and cancer-related pathways, such as insulin signaling pathway, WNT signaling pathway, renal cell carcinoma, pathways in cancer, MAPK signaling pathway, etc. Metabolic changes are prevalent in several human cancer tissues. The Warburg effect converts glucose to lactate, leading to cancer cell proliferation and increased biosynthetic demand [36]. ccRCC is considered to be a metabolic-driven disease process [37] and similar changes occur in the TME of ccRCC [38]. Multi-omic studies have identified several metabolic alterations in ccRCC, including the tricarboxylic acid cycle, pentose phosphate, and phosphoinositide 3-kinase (PI3K) pathways [39], and changes in specific intermediates of these pathways are associated with poor prognosis of patients [40, 41]. These findings suggest that metabolic alterations are associated with development and prognosis of ccRCC. Combined with our research results, we speculate that the up-regulation of BID may regulate the internal energy metabolism of ccRCC by hindering the insulin signaling pathway, thereby increasing the demand for tumor biosynthesis and leading to tumor progression. These dysregulated metabolism provide new insights into the development of ccRCC, as well as new ideas for treatment, intervention and diagnosis, in which BID may be a potential target. Moreover, a variety of signaling pathways also play vital role in the development of tumor. Fuertes et al. [42] found that non-classical WNT signaling promote colon tumor growth, chemotherapy resistance and tumor fibroblast activation. The up-regulation of WNT was observed in the progression of breast tumors and was associated with tumor drug resistance [43]. Wang et al. [44] found that activating WNT signaling can promote cell growth and invasion of ccRCC. Colic et al. [45] found that abnormal MAPK signaling transduction can provide therapeutic potential for the treatment of ovarian cancer. Li et al. [46] also demonstrated that MAPK/ELK1 axis can regulate BCL6 and promote the occurrence of KRAS-driven lung cancer. How BID promotes the occurrence and progression of ccRCC through the WNT signaling pathway or MAPK signaling remains to be further studied. These potential regulatory signaling pathways may provide some new ideas and insights for the future treatment of ccRCC.

Current studies have revealed that macrophages M1 and T cells CD8 generally exhibit tumor suppressive effects, while macrophages M2 and Tregs have immunosuppressive effects that promote tumor growth and metastasis [47]. This proves that the composition of immune cells in TME affects the effectiveness of tumor therapy. In this study, we found that the expression levels of BID was positively correlated with the TME score of ccRCC patients, and had a greater effect on the immune score. The tumor tissues with BID high expression may have more immune cells and stromal cells, which preliminarily reveal the significant effect of BID on the composition of TME. In addition, 10 kinds of immune cells in TME were related to the expression of BID, including T cells CD8, Tregs, macrophages M0 and T cells follicular helper, which were closely related to tumor. Noyes et al. [48] found that tumor-associated Tregs hinders anti-tumor immunity by promoting T cell dysfunction and limiting the clonal diversity of tumor-infiltrating CD8 + T cells. Studies by Zhu et al. [49]have shown that cancer cells often recruit Tregs and construct inhibitory TME through their anti-tumor immunity. Picard et al. [50] recently found that T cells CD8 producing IL-17A promote the development of pancreatic ductal adenocarcinoma by inducing inflammatory cancer-associated fibroblasts. Sanmamed et al. [51] also find that depleted T cell CD8 subsets expand and inhibit cancer immunotherapy in TME. In our study, highly invasive Tregs is associated with poor prognosis, which is consistent with previous studies. However, there was no correlation between the infiltration levels of T cells CD8 and the prognosis of patients. The results of ssGSEA also indicated that the samples with BID high expression had higher immune cell infiltration score and immune pathway activity, which once again revealed the complexity and diversity of immune infiltration of TME in ccRCC. Based on these results, we believe that BID is closely related to the immune microenvironment, and may mediate and regulate these immune infiltration characteristics.

ICIs play a crucial role in the treatment of ccRCC. We found that 4 immunostimulatory molecules and 5 immunosuppressive molecules were significantly correlated with the expression of BID, such as PD-1, LAG3, and CTLA4. Interestingly, the expression of these immune checkpoints was obviously up-regulated in the samples with BID high expression. We speculate that BID may participate in the regulation of immune checkpoints expression in ccRCC tissues. However, this regulation is complex because it affects both immunostimulatory molecules and immunosuppressive molecules, which needs to be further explored in the future. PD-L1 is the most promising biomarker of response to ICIs in most malignant tumors, but its predictive value in ccRCC is controversial. Mori et al. [52] showed that ccRCC patients with PD-L1-positive benefited more from combinations of ICIs than those with PD-L1-negative. However, the results of the CHEKMATE-025 [7] indicated that the improvement in OS in refractory RCC was consistent regardless of PD-L1 expression. Therefore, we also need to explore other reliable biomarkers for predicting immunotherapy response.

We found that samples with BID high expression possessed higher TIDE scores, predicting that such patients may benefit less from treatment of ICIs. We noticed that the samples with BID high expression had a higher degree of T cell infiltration, but also had a higher score of T cell dysfunction, which indicated that ccRCC mainly exerted immune escape by inducing T cell dysfunction. We also observed that the MSI of samples in BID low expression group was higher, which may explain the difference in prognosis of patients. TMB is also a potent predictor of response to ICIs [53]. We found that TMB was positively correlated with the expression of BID in ccRCC tissues. The samples with high expression of BID also had higher TMB, but the prognosis was worse. This revealed a close relationship between BID expression and TMB. However, ccRCC usually carries low to moderate mutation burden, and the efficacy of this biomarker in ccRCC needs more research. Somatic mutation is another important factor in tumorigenesis and development. We found that the most common type of somatic mutation in ccRCC samples was missense mutation, which was similar to that in most tumors. Notably, VHL mutation and PBRM1 mutation were the most common in two groups, revealing the ubiquitous gene mutation properties in ccRCC. However, VHL mutation, SETD2 mutation and BAP1 mutation had mutational differences of up to 10% between the two groups, suggesting that differential expression of BID mediates differential tumor mutational signatures. These results demonstrate that BID may affect the tumor immune microenvironment through multiple pathways, which provides a new perspective for exploring whether BID can be used as a novel therapeutic target and a predictor of immunotherapy response in ccRCC.

We have to admit that there are some limitations in our research. Firstly, the data we analyzed came from public databases, which may need to be validated in more prospective cohorts in the future. Secondly, our research preliminarily shows that BID may mediate and regulate the characteristics of immune infiltration in TME, but the specific mechanism and pathway still need more in-depth experimental researches.

## Conclusions

Our study have proved that BID is highly expressed in ccRCC tissues and is a reliable prognostic biomarker. The prognostic model based on BID can accurately predict the prognosis of ccRCC patients. BID is obviously related to the characteristics of immune infiltration in TME and may be a potential target for immunotherapy in patients with ccRCC.

## Data Availability

The datasets analysed during the current study are available in the TCGA database (https://portal.gdc.cancer.gov/), ICGC database (https://dcc.icgc.org/), GEO database (https://www.ncbi.nlm.nih.gov/geo/), UCSC Xena database (https://xenabrowser.net/), MSigDB database (http://www.gsea-msigdb.org/gsea/index.jsp, Hallmark pathways), TIDE website (http://tide.dfci.harvard.edu), and HPA database (https://www.proteinatlas.org/).

## Abbreviations

ccRCC: Clear cell renal cell carcinoma
RCC: Renal cell carcinoma
BID: BH3 interacting domain death agonist
TCGA: The Cancer Genome Atlas
ICGC: International Cancer Genome Collaboration
GEO: Gene Expression Omnibus
TME: Tumor microenvironment
OS: Overall survival
PFS: Progression free survival
ROC: Receiver operating characteristic curve
AUC: Area under the curve
ICIs: Immune checkpoint inhibitors
PCA: Principal component analysis
GSEA: Gene set enrichment analysis
ssGSEA: Single sample gene set enrichment analysis
TMB: Tumor mutation burden
Tregs: T cells regulatory
TIDE: The Tumor Immune Dysfunction and Exclusion
HPA: Human Protein Atlas
